# Role of Oxidants in Metal Extraction from Sulfide Minerals in a Deep Eutectic Solvent

**DOI:** 10.1021/acsomega.4c01052

**Published:** 2024-03-14

**Authors:** Ehsan Bidari, Chandra Widyananda Winardhi, Jose Ricardo da Assuncao Godinho, Gero Frisch

**Affiliations:** † Institut für Anorganische Chemie, Technische Universität Bergakademie Freiberg, 09599 Freiberg, Germany; ‡ Helmholtz Zentrum-Dresden Rossendorf, Helmholtz Institute Freiberg for Resource Technology, 09599 Freiberg, Germany

## Abstract

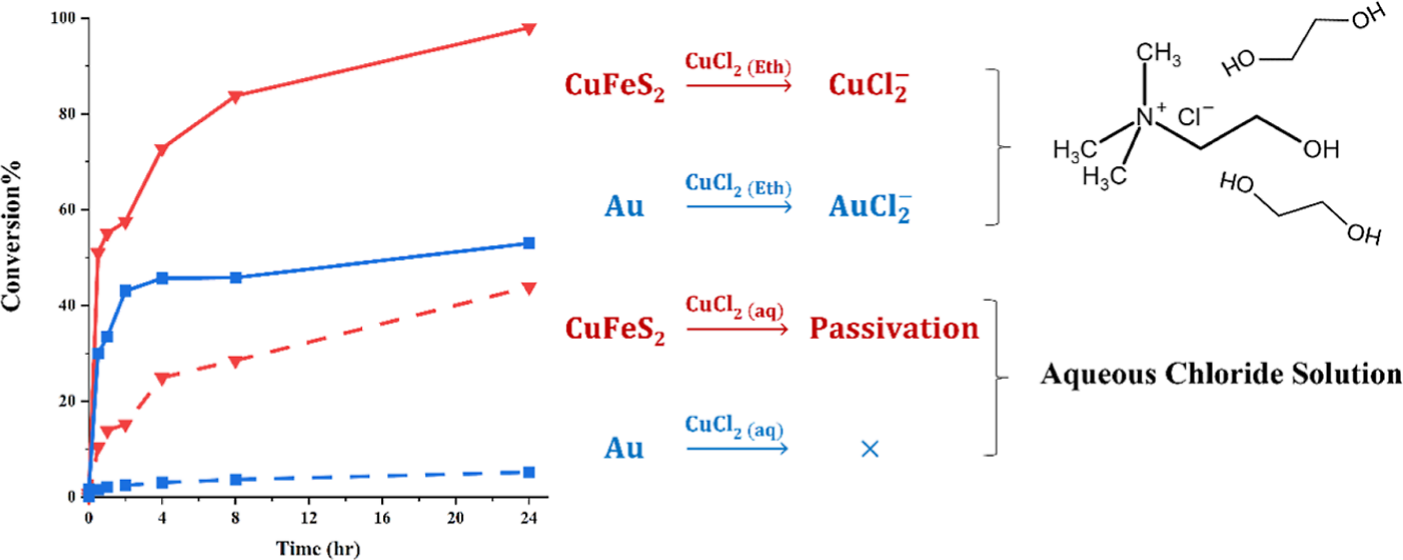

Metallurgical applications of deep eutectic solvents (DESs), known as ionometallurgy, have received significant research attention in recent years. While many studies claim that DESs are generally green and enhance process efficiency, others believe that industrial applications of ionometallurgy are generally not viable. Here, we report on leaching experiments of a sulfide flotation concentrate using ethaline, a chloride-based DES, in the presence of common oxidants. Following a mineral-based approach, we compare results with those obtained from aqueous chloride solutions to assess the influence of the leaching medium. We aim to contribute to a basic understanding of key differences between DESs and aqueous solutions and hope that this will help to make informed decisions about the suitability of DESs for leaching applications. Experiments were performed on a feed concentrate comprising a mixture of sulfide minerals along with substantial concentrations of Au, Ag, and Te. We found similar leaching behaviors for ethaline and aqueous solutions in nonoxidative leaching. However, when oxidizing agents were introduced, ethaline exhibited higher leaching efficiencies. Notably, the oxidation rate of pyrite in ethaline was very low, while chalcopyrite exhibited high oxidation rates. Furthermore, the results highlight significant variations in leaching rates depending on the type of oxidant, with the highest rate observed for I_2_, followed by CuCl_2_, and FeCl_3_. H_2_O_2_ and O_2_ were less effective. The leaching of gold–silver tellurides was possible in ethaline. This could be of particular significance, given that Au–Ag–Te compounds pose challenges in conventional cyanide treatment.

## Introduction

Sulfide minerals are the major source of world supplies of a very wide range of metals and are economically the most important group of ore minerals.^1^ The most processed sulfide minerals are pyrite (FeS_2_), chalcopyrite (CuFeS_2_), galena (PbS), and sphalerite (ZnS) since they are the main resources for refractory gold, copper, lead, and zinc, respectively.^2^

Pyrometallurgy is the main treatment method for sulfide minerals and demands high energy consumption and capital costs. Furthermore, pyrometallurgy faces environmental issues regarding the production of high volumes of CO_2_ and SO_2_. On the other hand, hydrometallurgical processes are generally suffering from the slow leaching rates for sulfide minerals. Galena was found to be readily dissolved in FeCl_3_ media, but chalcopyrite undergoes a more complex and slow reaction, and pyrite remains largely unaffected.^3^ The low leaching rate of chalcopyrite was related to the formation of a sulfide layer that is less reactive than the bulk of mineral.^4^ While the structure and composition of this surface layer remain a matter of debate, it has been found that passivation can be affected by solution pH, complexation agents, and the electronic structure of sulfide minerals.^5^ It has been reported that the presence of chloride ions improves the leaching efficiency by mitigating the formation of the passive layer but still does not completely prevent it from forming.^6^ The advantages of chloride systems are related to the higher solubilities of metal cations, improved oxidation of ferrous ions, and faster leaching rate compared with sulfate systems. Furthermore, in chloride solutions, elemental sulfur forms rather than sulfate during the oxidation of sulfide minerals.^7−9^ Efficient leaching of sulfide concentrates in aqueous chloride solutions requires acidic, oxidizing leach media and elevated temperatures. Considering the beneficial effect of chloride ions on the leaching of sulfide minerals, the application of chloride-based organic solvents as leaching media may bring about new advantages over aqueous systems.

Using nonaqueous solutions for the extraction of metals from ores, known as solvometallurgy or ionometallurgy, is a development aiming at the sustainable low-temperature recovery of valuable metals in ionic liquids (ILs) or deep eutectic solvents (DESs).^10^ DESs are binary or ternary mixtures with a deep melting point depression. They are usually obtained by the complexation of a quaternary ammonium salt with a metal salt or hydrogen bond donor.^11^ DESs are commonly recognized as a class of ILs due to their shared key features such as high thermal stability, low volatility, minimal vapor pressures, and adjustable polarity. In contrast, DESs are generally more cost-effective, biodegradable, nontoxic, and simpler to prepare than ILs.^12^ These characteristics make DESs promising candidates for leaching purposes.

Several advantages have been listed for ionometallurgy over traditional methods including limited consumption of water, limited generation of wastewater, the possibility of a single-step leaching-solvent extraction, more selectivity, and avoiding the formation of a passive layer and silica gel. In this way, ionometallurgy has been suggested as a promising method to develop near-zero-waste metallurgical processes, with levels of energy consumption that are much lower than those of the pyrometallurgical processes.^13^ While previous publications may have often been too optimistic about industrial applications in this field, recently a list of disadvantages has been published implying that innovations in hydrometallurgy based on ionometallurgy are not possible. This list highlights high viscosity, limited chemical stability, difficulties with recycling and reuse, and minimal added value compared to state-of-the-art hydrometallurgical processes as the main drawbacks of ionometallurgy.^14^

We believe that both approaches, “green game-changer” and “no worth looking into”, are misleading since they have been built on undue generalizations of DES properties and are not task-specific. The flexible architecture of DESs makes it possible to overcome some drawbacks. On the other hand, added value needs to be evaluated for each specific treatment task. Such an evaluation is difficult for the leaching of minerals because the underlying chemistry and key differences between ionic and molecular solvents remain largely unknown.

Ionometallurgical leaching of sulfide minerals has been considered in both neutral and ionic solvents. The main research focus in this area has been on leaching of chalcopyrite. It has been reported that chalcopyrite could be leached efficiently in a FeCl_3_–ethylene glycol (EG) solution forming FeCl_2_, CuCl, and solid elemental sulfur.^15^ Mixture of ILs and aqueous solutions has also been used for the leaching of chalcopyrite.^16^ Moreover, it has been noted that the addition of Cl^–^ into ILs enhances the dissolution of chalcopyrite due to the enhanced proton activity and complexation of Cl^–^ with copper ions giving a catalytic effect.^17^ A significant gap in the existing research is the lack of investigation into kinetic parameters for a meaningful comparison of ionometallurgy to aqueous leaching. One study that did investigate kinetic parameters demonstrated similar leaching efficiency and reaction mechanisms when comparing atmospheric leaching of chalcopyrite in both aqueous H_2_SO_4_ and acidic ILs (imidazolium-based), after correction for pH differences.^18^ This finding underscores the necessity for a more comprehensive research approach to assess the efficacy and potential of ionometallurgical processes.

Using DESs as leaching media is based on the promising results of imidazolium ILs. DESs provide similar chemical properties but, in most cases, are much cheaper and often environmentally less problematic. The high solubility of base and precious metals in chloride-based DESs^19,20^ allows their application for the extraction of metals. Previous research has suggested that chalcopyrite concentrates can be dissolved in a nonredox process in ethaline, a chloride/EG DES, under ambient conditions. Cu and Fe are leached without changing their oxidation state, without solvent pH change, and stabilized as a chloride complex with no evidence of passivation.^21^ In the presence of urea, dissolved Cu has been found in a mixed chloride/O- or N-donor coordination.^22^ It is important to note that while these studies show promising results, they lack sufficient characterization to prove their assumptions regarding passivation and leaching mechanisms.

Limited information is available concerning leaching of other sulfide minerals in DESs. While the extraction of zinc and lead from oxide resources such as furnace dust has been investigated,^23,24^ the leaching behavior of sulfidic minerals of zinc and lead has not been investigated in detail yet. Mineral-based studies of sulfide and precious metal leaching in the DES-iodine system have demonstrated that pyrite remains intact during leaching. It has been also mentioned that while the leaching rate of chalcopyrite is low, galena undergoes rapid dissolution.^25,26^ These results contradict the idea of nonredox leaching of chalcopyrite, although the main focus was on simulating mineral leaching in DES, and the effect of oxidant has not been discussed.

In the case of precious metals, it has been reported that oxidative leaching in IL media improves the extraction of silver, while gold extraction was found similar to that achieved in the aqueous system.^27−29^ In DES media, it has been demonstrated that electrum, native Te, and tellurobismuthite are soluble by oxidation with iodine.^26^ This study shows very interesting results but lacks a comparison with the aqueous system and has been conducted only with very short leaching times.

In summary, despite promising reports on the positive impact of using DESs in the leaching of sulfide minerals, fundamental questions, such as enhanced efficiency compared to aqueous solutions and the necessity of employing oxidizing agents, remain unanswered. More in-depth issues, such as the reaction mechanism, alterations in passivation, and kinetic analysis, are notably absent. The absence of such information complicates the assessment of DESs as leaching media.

In this study, we investigated the influence of using oxidizing agents on the dissolution of sulfide minerals. Currently, there are limited studies available regarding the use of I_2_ as an oxidant in DES, while more economical and industrially viable agents such as oxygen, Fe^3+^, and Cu^2+^ remain unexplored. The significance of this matter becomes more apparent when considering reports related to changes of redox potential in DES compared to aqueous solutions.^30,31^

Ethaline, a well-known chloride-based DES, was used as the leaching medium in this study. Ethaline belongs to the type III DESs formed from a hydrogen bond donor and a hydrogen bond acceptor. Such DESs have been of interest for hydrometallurgical applications due to their ability to solvate a wide range of transition metal species.^11^ Ethaline is an almost neutral DES,^32^ consequently, the dissolution behavior of sulfide minerals and the impact of oxidants can be assessed independently of solution acidity. Furthermore, the lower viscosity of ethaline compared to many other chloride-based DESs allows for enhanced mass transfer and conducting the investigations in a chemical reaction-controlled regime. The results are interpreted in a mineral-based way, and the obtained results in ethaline have been compared with those of aqueous systems with comparable concentrations of chloride ions and obtained under similar operational conditions. With this study, we aim to highlight key differences between DES and aqueous media, which may help to point out opportunities and assess the viability of industrial applications in the future.

## Material and Methods

### Chemicals

All of the chemicals used in the present work were of analytical grade.

### Preparation of Ethaline

Ethaline was prepared by mixing choline chloride [ChCl (C_5_H_14_NOCl), Sigma-Aldrich, ≥98%] and ethylene gylcol [EG (C_2_H_6_O_2_), Sigma-Aldrich, ≥98%] at a molar ratio of 1:2 and 343 K until a colorless, transparent, and homogeneous solution was formed. The water content of ethaline was determined to be 0.1–0.3% using Karl Fischer titration.

### Feed Sample

A sulfide concentrate (particle size < 125 μm) containing precious metals from the Cononish gold mine in the Scottish Highlands was used in this study. Mineralogical characterization of the concentrate was carried out using X-ray diffraction (XRD, Bruker D8 discover) (Figure S1) as well as mineral liberation analyzer (MLA, FEI Company, Hilsboro, OR, USA) for quantitative analysis. The results are summarized in Table 1 showing that the concentrate mainly consists of pyrite, quartz, chalcopyrite, galena, and sphalerite.

**Table 1:** Mineralogical Composition of the Feed Concentrate

phase	chemical formula	content %
pyrite	FeS_2_	55.02
chalcopyrite	CuFeS_2_	6.94
galena	PbS	5.52
sphalerite	ZnS	1.48
quartz	SiO_2_	19.46
electrum	AuAg	0.02
acanthite	Ag_2_S	0.01
hessite	Ag_2_Te	0.05
petzite	Ag_3_AuTe_2_	0.04
others	mainly silicate and carbonates	11.46

Elemental analysis was performed through microwave acidic digestion (HCl + HNO_3_ + HF) followed by inductively coupled plasma mass spectrometry. The results show that Au, Ag, and Te are the main precious metals in the sample (Table 2). MLA revealed that electrum, acanthite, hessite, and petzite are the main minerals carrying the precious metals. A close association between precious metals and sulfide minerals was also identified. It was found that petzite, hessite, acanthite, and electrum are hosted by pyrite, galena, and alkali feldspar. Electrum was found to be associated with several silicates and sphalerite. Poor liberation was noted only for petzite.

**Table 2:** Elemental Composition of Precious Metals in the Feed Concentrate

element	Ag	Te	Au
conc. (ppm)	1268	460	211

Four of the most common sulfide minerals present in ores are found in the concentrate, which makes it a suitable sample for leaching studies, where the dissolution behavior of each mineral can be followed.

### Leaching Tests and Analysis

Leaching experiments were performed in a batch reactor that was mechanically stirred at 300 rpm. The tests were carried out with a solid/liquid ratio of 2:100 ensuring that external mass transfer is not the rate-controlling step. Experiments were done in ethaline and aqueous chloride solutions in the presence of different oxidants of redox equivalent concentrations, namely, 0.2 M FeCl_3_, 0.2 M CuCl_2_, 0.1 M I_2_, 0.1 M H_2_O_2_, and O_2_ (purged into solution during leaching time). Aqueous leaching solutions were prepared at the same chloride ion concentration as that of ethaline (4.3 M) by the addition of NaCl. 0.1 M HCl was also added to the aqueous systems to prevent the precipitation of metals as hydroxides. In this way, the leaching efficiencies of sulfide minerals in ethaline can be compared to those of common chloride leaching in an acidic aqueous solution.

During leaching experiments, samples were withdrawn periodically and filtered (at the same temperature as leaching experiments), and the filtrates were analyzed using ICP-OES. The progress of the leaching reactions was monitored by the calculation of conversion values. Fe, Cu, Pb, and Zn concentrations were used for the calculation of the conversion of pyrite, chalcopyrite, galena, and sphalerite, respectively. Pyrite conversion was calculated after correction of the amount of Fe that leached out from chalcopyrite. Considering the general leaching reaction as
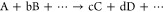
1

The conversion percentage of mineral A, *X*_A_, defines the number of moles of A that have reacted per mole of A fed to the system^33^
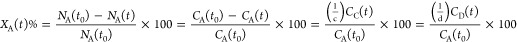
2where *N*_A_(t_0_) is the initial amount of A in the reactor, and *N*_A_(t_t_) is the amount of A in the reactor at the time *t*. Since the reactor volume is constant in a batch system, the mole values can be replaced by the concentrations (*C*_A_). Using stoichiometric coefficients, the concentration of the minerals can be related to the concentration of reaction products.

At the end of the leaching experiments, the solid and liquid phases were separated by filtration for further analysis. The solid residues were analyzed by XRD and scanning electron microscopy (SEM, Tescan Vega SB) with energy-dispersive X-ray analysis (EDX). Leaching solutions were analyzed by using UV–vis spectroscopy (Jasco V670) and open circuit potential (OCP, Gamry) measurements. Potential measurements in aqueous solutions were referenced against a AgCl/Ag electrode (3 M KCl). For DES samples, a silver wire immersed in a 0.1 M solution of AgCl in ethaline was used as a reference electrode. It is vital to note that using different electrolytes may cause a shift in reference potentials unless referenced against an internal standard.^34^ Potentials are, hence, not directly comparable between aqueous solutions and DESs.

It was not possible to repeat leaching experiments often enough to obtain statistically relevant numbers for every single data point. Errors of quantitative leachate analysis were hence evaluated as follows. Three independent leaching experiments carried out under the same conditions (80 °C, 0.2 M FeCl_3_, 24 h) in ethaline were used for the error estimation. The typical errors were determined to be 5–10%. The relative standard deviations of ICP-OES analysis were significantly lower than these errors and were hence not further considered.

## Results and Discussions

### Leaching

Leaching experiments were conducted both in the absence (nonoxidative) and presence (oxidative) of oxidants under atmospheric conditions.

To gain a better understanding of the impact of oxidants, the OCP of the pregnant leaching solution was measured after 24 h of leaching (Table 3). OCP values for aqueous solutions appear to be higher than those for ethaline. However, this potential shift may arise from differences in liquid junction potential and reference electrode design. We hence only compare the potentials of samples using the same solvent. Table 3 demonstrates broader potential differences between leaching solutions in ethaline compared to those in an aqueous solution. This allows for the control of potential in a desired range using common oxidants. Such control is important, particularly in the leaching of minerals like chalcopyrite, which are prone to potential-related passivation.^35^Table 3 also indicates low OCP values in ethaline for nonoxidative, 0.1 M H_2_O_2_, and O_2_ purging leaching solutions. These results imply the limited solubility of oxygen in ethaline. Higher OCP values in the aqueous systems could further be attributed to oxygen reduction in an acidic solution, a phenomenon that may not occur in ethaline due to the low concentration of H^+^ ions.

**Table 3:** OCP of Leaching Solutions [mV (Ag/AgCl)]

oxidant	ethaline	aqueous
	147	531
0.2 M FeCl_3_	511	544
0.1 M I_2_	368	435
0.2 M CuCl_2_	502	599
0.1 M H_2_O_2_	50	
O_2_	111	

**Figure 1:**
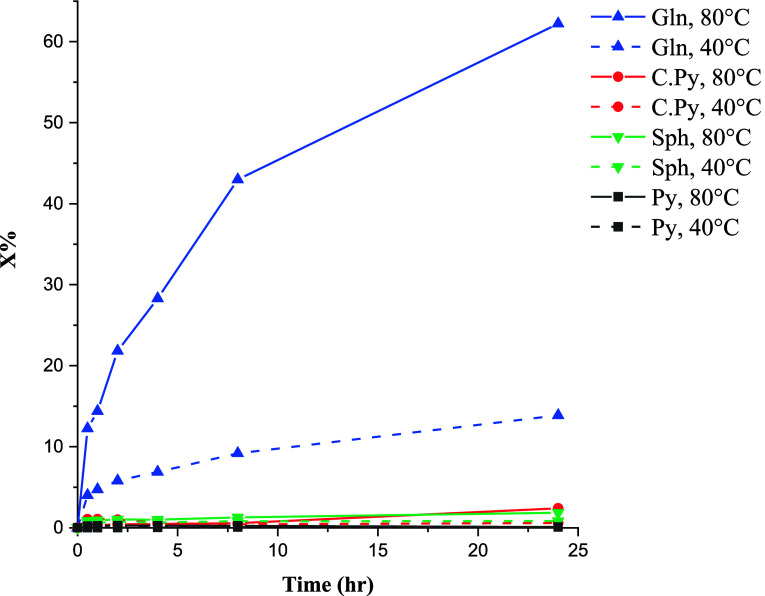
Conversion of pyrite, sphalerite, chalcopyrite, and galena in ethaline at 40 and 80 °C over time.

The leaching behavior of sulfide minerals in ethaline without the addition of oxidants is shown in Figure 1 at 40 and 80 °C. It is clear that without oxidizing agents only galena readily dissolves in ethaline. Very low conversion values were observed for other minerals which may be related to the oxidation by residual oxygen. It can be concluded that galena undergoes a nonoxidative reaction, but the dissolution of other sulfides needs oxidative leaching. On the other hand, it can be seen that temperature has a significant effect on the leaching yields. This is in agreement with the high activation energy of sulfide minerals which is well established in the case of both aqueous^36^ and IL^37^ media. Accordingly, 80 °C was selected for further experiments.

**Figure 2:**
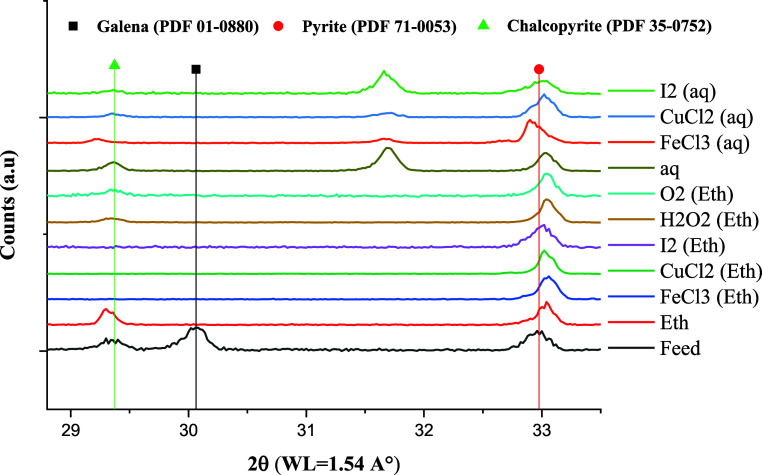
XRD pattern of feed samples and residues after 24 h leaching in ethaline at 80 °C (the whole range scan is provided in Supporting Information, Figure S1).

XRD analysis of leaching residues (Figure 2) indicates that pyrite remained almost intact, even in the presence of oxidizing agents. However, the characteristic reflection of galena disappeared in all cases. Given that the initial concentration of galena was 4.7% and over 60% dissolved, the final concentration expected in the solid is less than 2%, which approaches the detection limit of the XRD under these conditions. The intensity of the reflections of chalcopyrite varies by the oxidant, showing a more complex leaching behavior. Subsequent sections will provide further elaboration on the leaching behavior of each sulfide mineral and precious metal.

### Galena

**Figure 3:**
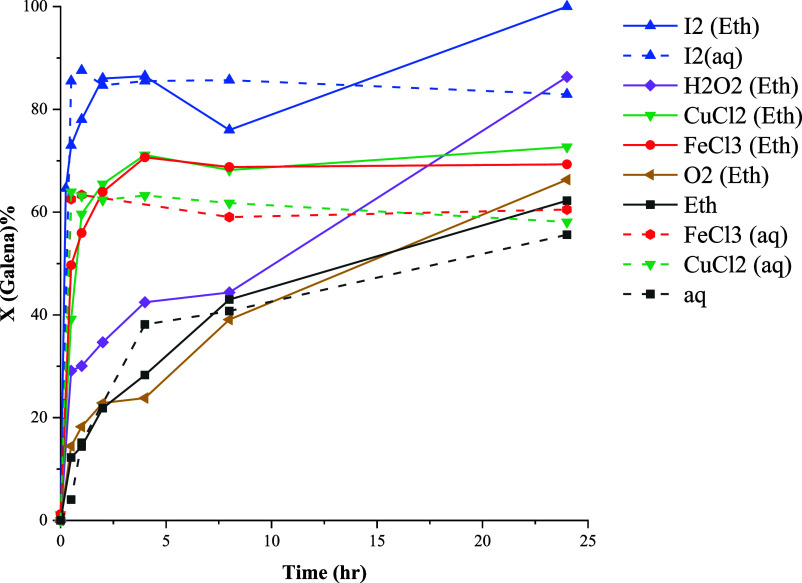
Effect of oxidizing agents on the conversion of galena at 80 °C.

The conversion curves of galena leaching in ethaline and aqueous systems are presented in Figure 3. The dissolution of galena was found to be feasible under both nonoxidative and oxidative conditions. Leaching efficiencies between ethaline and aqueous systems exhibit a notable similarity, albeit with slightly higher conversion values in the former case. Figure 3 illustrates that the presence of oxidizing agents enhances the initial rate of leaching, with significantly higher initial rates observed in systems employing FeCl_3_, CuCl_2,_ and I_2_ compared to H_2_O_2_ and O_2_.

Distinct leaching behavior was discerned when FeCl_3_, CuCl_2_, and I_2_ were used in the ethaline. In these instances, the rapid initial dissolution of galena was succeeded by an apparent decrease in lead extraction. This phenomenon, particularly pronounced in the case of I_2_ leaching in ethaline, suggests the precipitation of initially dissolved lead. Examination of leaching residues (Figure S2) revealed the presence of PbCl_2_ particles. The conversion rate experiences a subsequent increase in the leaching duration. This phenomenon may be attributed to the escalating water content within the ethaline system. This increase can occur via moisture adsorption or the gradual decomposition of ethaline due to esterification reactions.^38^

The results imply that galena dissolution may happen through both nonoxidative and oxidative pathways, with the leaching rate being notably accelerated in the latter scenario. It is proposed in the literature that in the aqueous system, galena dissolution can progress through reactions 3–5, corresponding to reductive, nonoxidative, and oxidative reactions, respectively.^8^
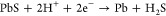
3
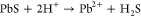
4
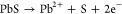
5

Reaction 3 postulates a reductive dissolution process leading to the formation of elemental lead. Given that lead was mainly found in the form of soluble ions within the solution, reaction 3 may not present the primary pathway for galena dissolution in ethaline. Furthermore, the OCP of the leaching solutions falls within the range of 50–510 mV (as indicated in Table 3), which supports the idea of nonreductive leaching of galena in ethaline.

**Figure 4:**
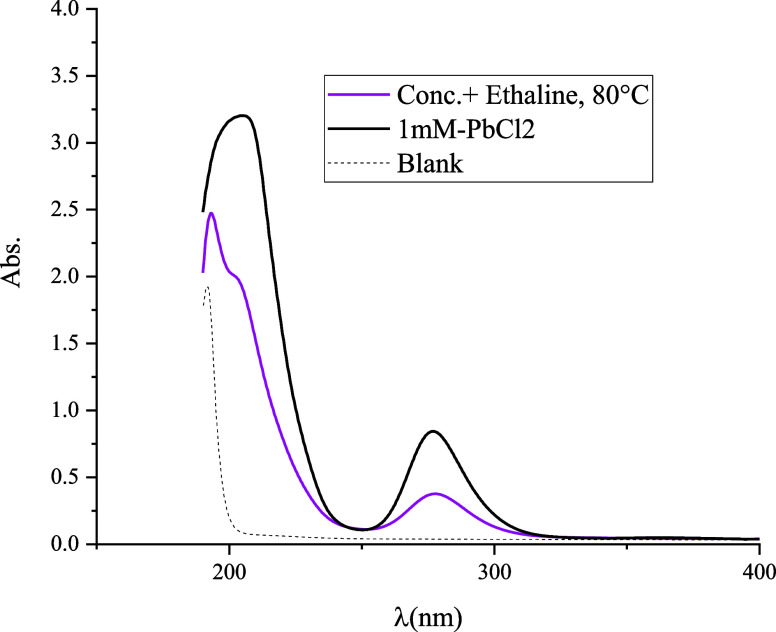
UV–vis spectra of concentrate leaching in ethaline at 80 °C compared to the model solution made of 1 mM PbCl_2_ in ethaline.

Conversely, reaction 4 represents a nonoxidative reaction applicable to both aqueous and ethaline systems, given the similarity in leaching behavior observed among these systems. The nonoxidative dissolution of galena aligns with chemical bonding information, wherein the bonding within galena has been described as predominantly ionic with minor covalent (or metallic) characteristics.^39^ To identify the lead species in ethaline, the UV–vis spectrum of the leaching solution was compared to that of a PbCl_2_ model solution (Figure 4). Table 4 presents the concentrations of various ions in the leaching solution following a 24 h leaching period in ethaline without the addition of any oxidants. The results reveal a high concentration of lead ions compared to other metals, suggesting that the solution’s composition is primarily attributed to the dissolution of galena. Consequently, the UV–vis spectrum of this system can be predominantly attributed to lead species.

**Table 4:** Elemental Analysis of PLS after 24 h of Leaching, 80 °C

element	Fe	Cu	Zn	Pb	S
conc. (ppm)	15.0	11.6	4.1	507.6	102.9

The spectra obtained from the both model and leaching solutions were similar, indicating the formation of the same lead species. The discerned peak at around 280 nm can be attributed to PbCl_4_^2–^ species.^20^

Hence, reaction 6 can be proposed to represent the nonoxidative reactions of galena
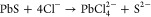
6

Sulfide ions may form HS^–^ or H_2_S in further steps
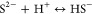
7
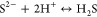
8

**Figure 5:**
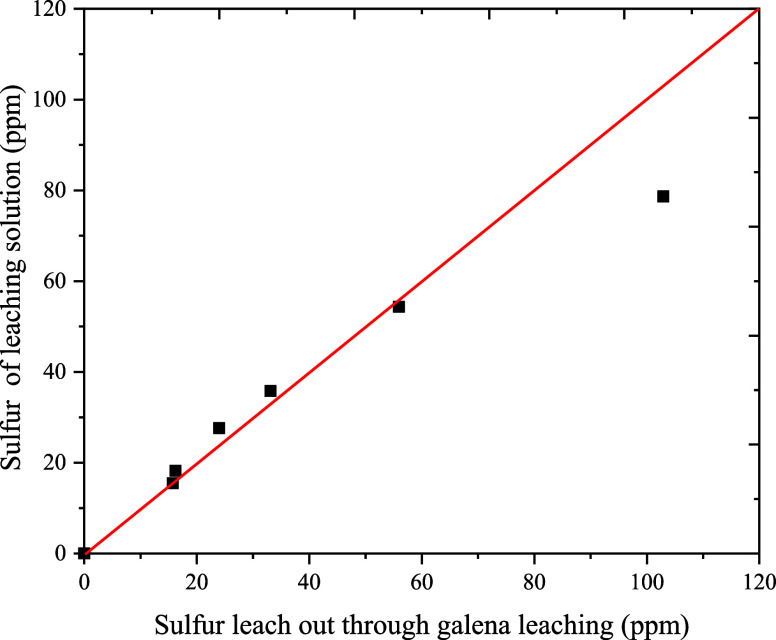
Comparison of the content of soluble sulfur in leaching solution (ethaline, 80 °C) with those calculated from galena conversion over time.

Figure 5 compares the dissolved sulfur content in the leaching solution (without oxidants) to the theoretical amount expected to be leached through galena conversion (calculated stoichiometrically).

During the initial 8 h, a strong agreement exists between the measured and calculated data, indicating that galena dissolution yields soluble sulfur species, as represented by reaction 7. A deviation becomes evident in the 24 h leaching data, suggesting that a portion of S^2–^ undergoes evolution into H_2_S (reaction 8).

In the presence of oxidant agents, S^2–^ may oxidize to elemental sulfur
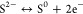
9

The leaching results illustrate that the rate of oxidative leaching in ethaline is faster than the nonoxidative one. This can be due to the slow mass transfer of HS^–^ or H_2_S from the surface of galena or a low proton concentration.

### Pyrite

**Figure 6:**
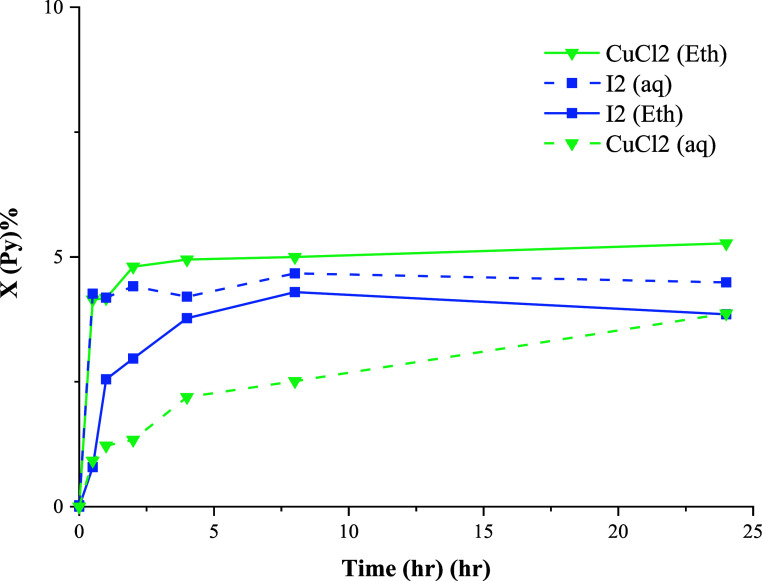
Effect of oxidizing agents on the conversion of pyrite at 80 °C.

The conversions of pyrite using CuCl_2_ and I_2_ are presented in Figure 6. Very low conversion values (*X* < 1%) were observed in other cases. These results agree with XRD (Figure 2) and SEM/EDX analyses in which pyrite particles were identified with clean surfaces without any proof of oxidation. The half-reaction of pyrite oxidation in the acidic chloride system can be described by reactions 10 and 11(40)
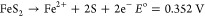
10
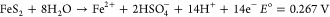
11

According to the formal potential values, O_2_/H_2_O, Fe^3+^/Fe^2+^, and I_2_/I^–^ couples can oxidize pyrite.^41^ In a chloride system, oxidation can also be accomplished by Cu^2+^/Cu^+^ according to reaction 12(40)
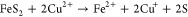
12

It is vital to note that the presence of Cl^–^ ions can strongly influence the redox potential of these couples. It has been found that a high concentration of Cl^–^ leads to a reduction in the formal potential of Fe^3+^/Fe^2+^ while that of Cu^2+^/Cu^+^ increases.^42^ This observation aligns with the data presented in Figure 6, demonstrating that pyrite exclusively undergoes the oxidative reaction in the presence of CuCl_2_ and I_2_, albeit with relatively slow reaction kinetics.

### Chalcopyrite

**Figure 7:**
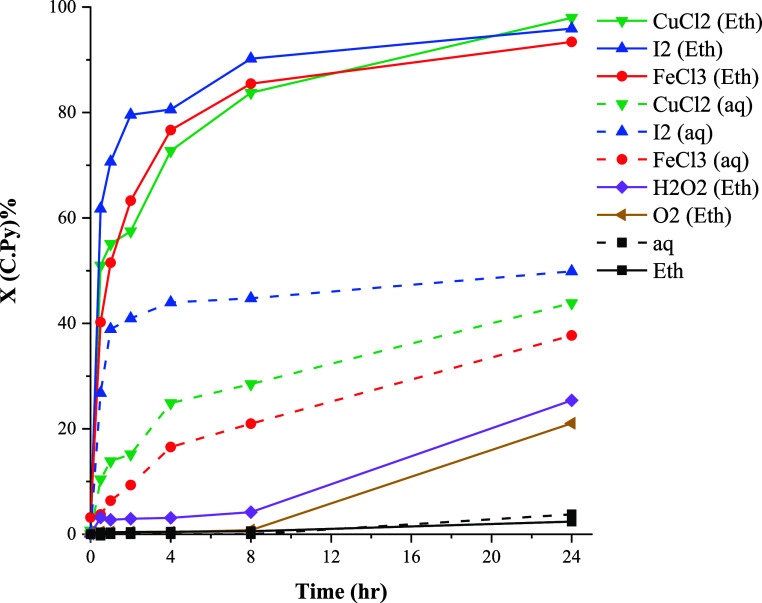
Effect of oxidizing agents on the conversion of chalcopyrite at 80 °C.

The results of chalcopyrite leaching (Figure 7) illustrate that, unlike galena, chalcopyrite does not readily undergo a nonoxidative reaction in ethaline or aqueous systems. Chalcopyrite has the formal oxidation states of Cu^I^Fe^III^(S^–II^)_2_.^43^ It has been suggested that Cu(I) and Fe(III) can keep their oxidation state in the leaching solutions of high chloride concentration while S^2–^ forms H_2_S, HS^–^, or will oxidize to S(s).^44−46^ Accordingly, the nonoxidative dissolution of chalcopyrite can be described by reactions 13 and 14
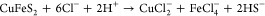
13
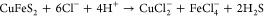
14

Nonoxidative dissolution of chalcopyrite has been found to occur slowly, primarily due to the slow diffusion of soluble species away from the mineral surface.^21,47^

It can be seen in Figure 7 that using oxidants improves the conversion of chalcopyrite in both aqueous solution and ethaline. This enhancement can be correlated with the oxidation of H_2_S, enhancing the diffusion of H_2_S away from the chalcopyrite surface. Consequently, this oxidation process significantly contributes to the overall reaction rate. It is generally accepted that elemental sulfur is the main product of chalcopyrite oxidation.^7^ Thus, the overall reaction of chalcopyrite leaching can be given by the combination of reactions 14 and 15 (in the case of using FeCl_3_ as the oxidant). Similar reactions can be performed with copper(II) ions or other oxidants.
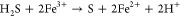
15

**Figure 8:**
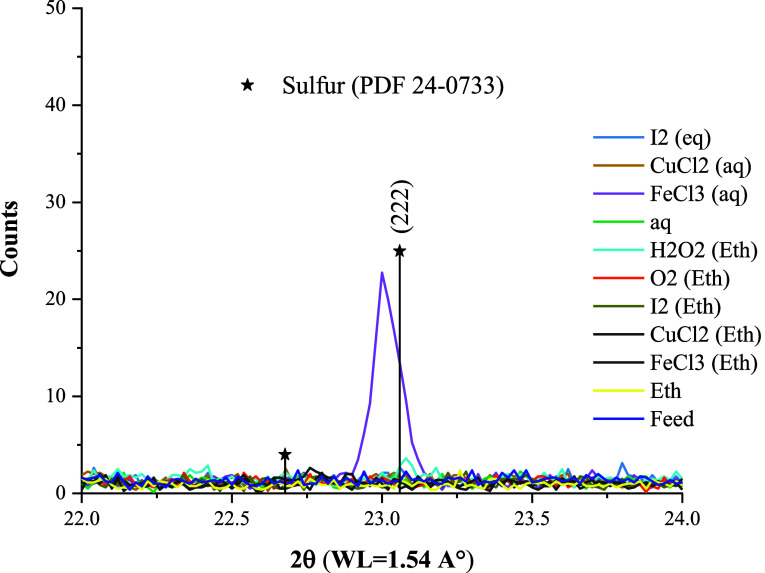
XRD pattern of residues after 24 h leaching in ethaline at 80 °C (the whole range scan is provided in Figure S1).

We could identify elemental sulfur using XRD analysis (Figure 8) only in the leaching residues of the aqueous system with 0.2 M FeCl_3_. Sulfur in other systems could form an amorphous precipitate or different solution species. Further investigations regarding sulfur speciation in ethaline seem necessary.

Figure 7 also shows that the leaching yield in ethaline is considerably higher than that of the aqueous solutions under similar conditions. Leaching of chalcopyrite in aqueous media was found to be retarded by passivation due to the formation of a relatively thin copper-rich polysulfide layer.^35^ It was reported that the formation of a passive layer may be hindered in nonaqueous solvents.^22^ There is no detailed information available regarding how nonaqueous media affect the leaching mechanism of chalcopyrite. The results also show that the rate of chalcopyrite leaching varies significantly depending on the oxidant. The highest recovery was observed in the case of CuCl_2_ followed by those of I_2_ and FeCl_3_. Conversely, H_2_O_2_ and O_2_ exhibited a lower effectiveness in facilitating chalcopyrite leaching.

### Sphalerite

**Figure 9:**
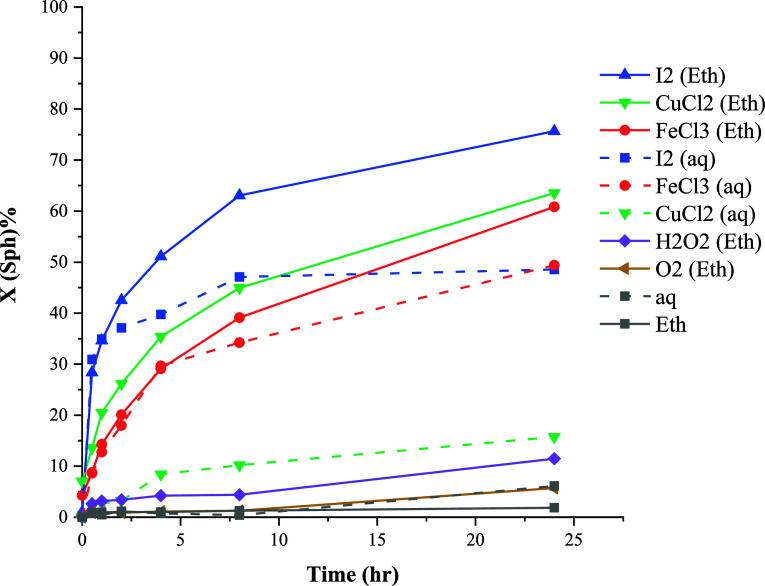
Effect of oxidizing agents on the conversion of sphalerite at 80 °C.

Figure 9 demonstrates that similar to chalcopyrite, sphalerite shows low leaching rates in the absence of oxidizing agents, as well as in the presence of H_2_O_2_ and O_2_. When FeCl_3_ is present, a nearly identical leaching yield was observed in both the aqueous solution and ethaline. Notably, a substantial enhancement was evident in ethaline when CuCl_2_ and I_2_ were utilized as the oxidants.

### Precious Metals

**Figure 10:**
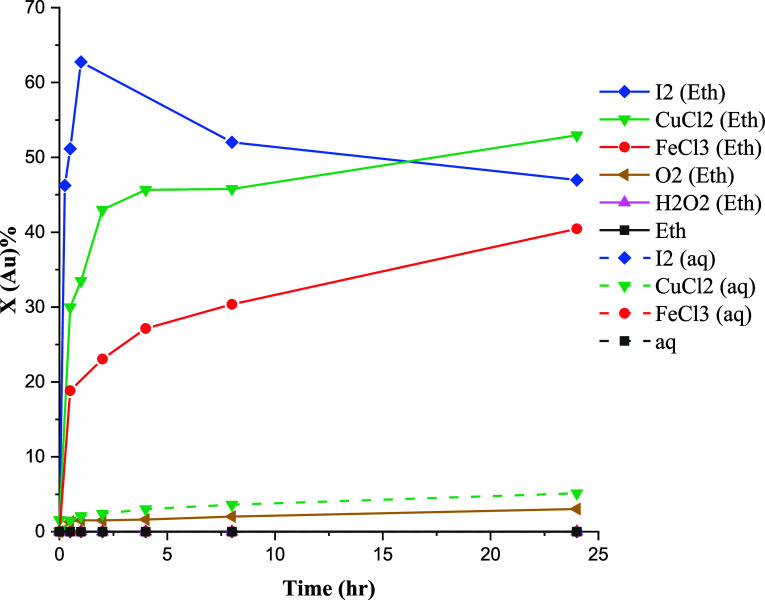
Effect of oxidizing agents on the conversion of gold at 80 °C.

**Figure 11:**
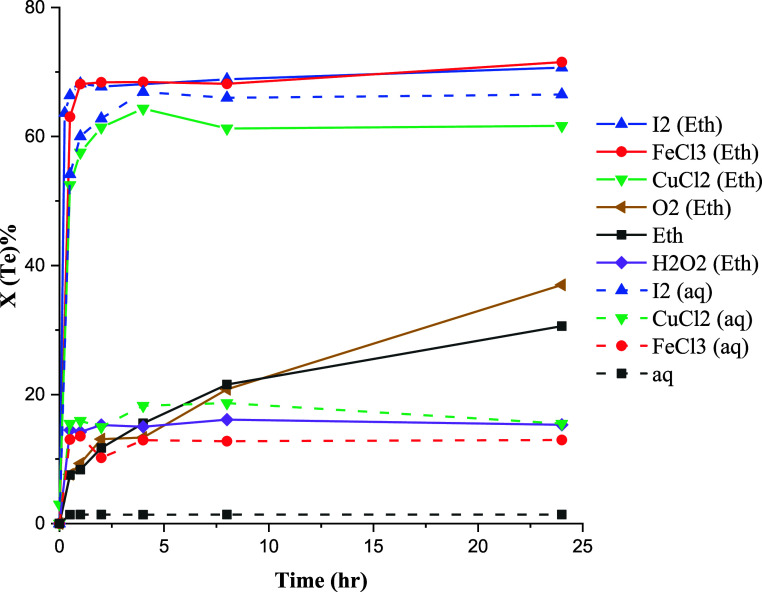
Effect of oxidizing agents on the conversion of tellurium at 80 °C.

**Figure 12:**
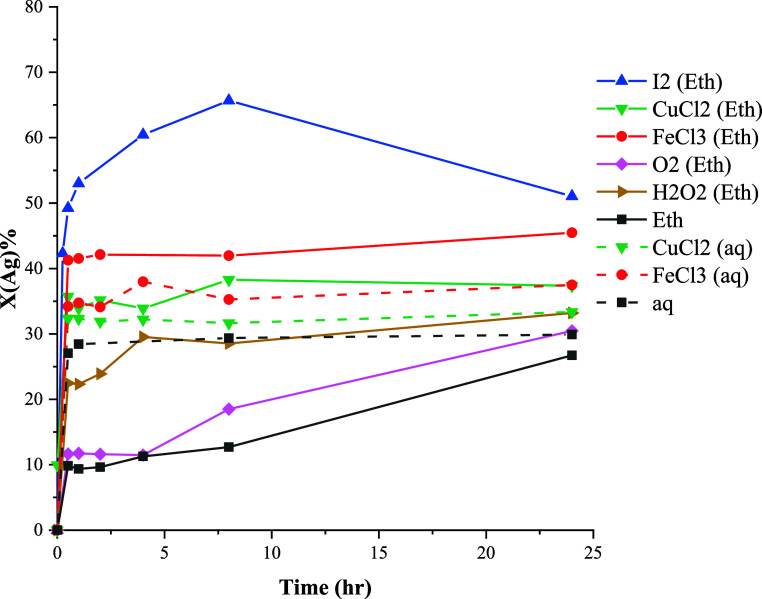
Effect of oxidizing agents on the conversion of silver at 80 °C.

The conversions of Au, Ag, and Te at 80 °C employing various oxidants are depicted in Figures 10–12. The results reveal a significant improvement in conversion values in ethaline compared with the aqueous solution for all precious metals, though a complete conversion was not achieved. In Figure 10, it becomes evident that Au predominantly remains insoluble in the aqueous solutions. However, in ethaline, conversion values exhibited improvements in the presence of FeCl_3_, CuCl_2_, and I_2_, with CuCl_2_ and I_2_ yielding higher leaching rates.

Characterization results (Table 1) have shown that Au is in close association with Te and Ag and forms compounds, such as Ag_3_AuTe_2_ and AuAg. Telluride minerals are soluble in acidic chloride media in the presence of moderate oxidants.^46^ Accordingly, the partial leaching of Au in the presence of oxidants (Figure 10) can be related to the dissolution of Ag_3_AuTe_2_. This observation aligns with the outcomes of Te leaching (Figure 11), where substantial conversion of Te was achieved under oxidative leaching conditions. The leaching of gold–silver tellurides in ethaline assumes particular significance, given that Au–Ag–Te compounds pose challenges in conventional cyanide treatment methods due to their refractory nature.

Another Au-bearing phase, electrum (AuAg), was found to exhibit oxidation behavior similar to that of native gold. The dissolution of gold in the aqueous chloride solution happens through reactions 16 and 17, forming Au^+^ and Au^3+^ species, respectively^46,48^
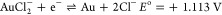
16
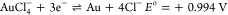
17

As a result, the oxidation of gold, at a rate suitable for industrial processes, necessitates high potentials and the utilization of strong oxidizing agents. Solution potential values (as presented in Table 3) indicate that gold dissolution would not occur at a practically viable rate in either aqueous solutions or ethaline. Conversely, the application of alternative halides, such as I_2_, can facilitate the rapid dissolution of gold, aligning with the predictions based on electrode potentials^46^
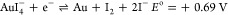
18

This condition appears to hold true in the case of I_2_ leaching in ethaline, where a noteworthy enhancement in the oxidation of both Au and Ag was observed (Figures 10 and 12).

Ag conversion curves show almost similar values for Ag extraction under all conditions except in the presence of I_2_ in ethaline. This behavior can be attributed to the leaching of ionic compounds, such as argentite (Ag_2_S) in chloride media. In the case of I_2_ leaching in ethaline, a significant increase in conversion, similar to that observed for Au, was found. In I_2_ leaching, after the fast initial leaching, the conversion values for both Au and Ag decreased over time. This may be attributed to the precipitation of Au and Ag iodide in ethaline, though no solubility data is currently available to evaluate this in ethaline.

## Conclusions

The results obtained in this study revealed enhanced oxidative leaching of sulfide minerals and precious metals in ethaline compared with analogous aqueous chloride solutions. A distinct behavior of the different oxidizing agents was also observed. Notably, CuCl_2_, FeCl_3_, and I_2_ demonstrated high effectiveness, whereas H_2_O_2_ and O_2_ exhibited slow reaction rates. I_2_ was identified as the most effective oxidant, exhibiting the highest efficiencies and leaching rates for the target minerals and metals.

Among sulfide minerals, a significant improvement was observed in the chalcopyrite leaching. Pyrite, as the main gangue mineral in the sulfidic concentrates, showed a very low leaching rate in ethaline, making selective leaching possible.

In the case of precious metals, Au was extracted efficiently in ethaline, while it remained intact in the aqueous solutions. Significant advantages were also found for the extraction of Ag and Te in ethaline. This holds particular importance given the refractory nature of Au–Ag–Te alloys with respect to conventional cyanidation methods.

Differences in the leaching performance between ethaline and aqueous solution could originate from two factors: enhanced redox properties of oxidants and changes in the sulfide leaching mechanism. ChCl provides a high concentration of Cl^–^ ions in ethaline. Therefore, the speciation of ions in the leaching solution will be governed mainly by the concentration of chloride ions. The absence of water is also effective in this regard since metal hydration may compete with chloride complexation.^49^ Accordingly, changes in redox behavior may occur between DES and aqueous chloride solutions.^50^ This is also supported by previous studies indicating variations in redox potentials between ethaline and standard aqueous systems.^30,51^ The leaching mechanism of sulfide minerals in DES is likely to be different. Available investigations in aqueous chloride media have often centered on the formation of a passive layer as the rate-controlling phenomenon. Our findings suggest the possibility of differing sulfur speciation in ethaline compared with aqueous solutions. This may affect the passivation of the sulfide minerals. It is important to note that there is a lack of available literature addressing how these mechanisms operate in ethaline.

Accordingly, we believe that there is still a significant scientific gap to the extent that one can assess the industrial application of DESs in hydrometallurgy. To overcome this deficiency, mechanistic studies to rationalize the observed distinct behavior of sulfide minerals in DESs have to be conducted. Rate equations to elucidate the effective parameters governing leaching in DES must be established. In this paper, we highlighted how common oxidants can affect the leaching. The same is required about the effects of acidity and water content. This knowledge is required as a basis for designing or selecting suitable DESs for leaching and avoidance of undue generalizations or trial-and-error approaches.

## Supporting Information Available

XRD patterns of feed samples and residues after 24 h leaching in ethaline at 80 °C and SEM/EDX analysis of solids precipitated from leaching solution after a week (PDF)
