# Supplementary material for: Role of Oxidants in Metal Extraction from Sulfide Minerals in a Deep Eutectic Solvent
Source: ACS Omega. 2024 Mar 14;9(12):14592–603. doi: 10.1021/acsomega.4c01052 (PMC10976405; doi:10.1021/acsomega.4c01052)
Supplement: Supplementary file 1 — ao4c01052_si_001.pdf [file ao4c01052_si_001.pdf]

The Role of Oxidants in Metal Extraction from Sulfide Minerals in a Deep Eutectic Solvent

Ehsan Bidari [1], Chandra Widyananda Winardhi [2], Jose Ricardo da Assuncao Godinho [2], Gero Frisch [1*]

1. Institut für Anorganische Chemie, Technische Universität Bergakademie Freiberg, 09599 Freiberg, Germany
2. Helmholtz Zentrum-Dresden Rossendorf, Helmholtz Institute Freiberg for Resource Technology, 09599 Freiberg, Germany

* Corresponding Author, E-mail: frisch@tu-freiberg.de

Supporting Information

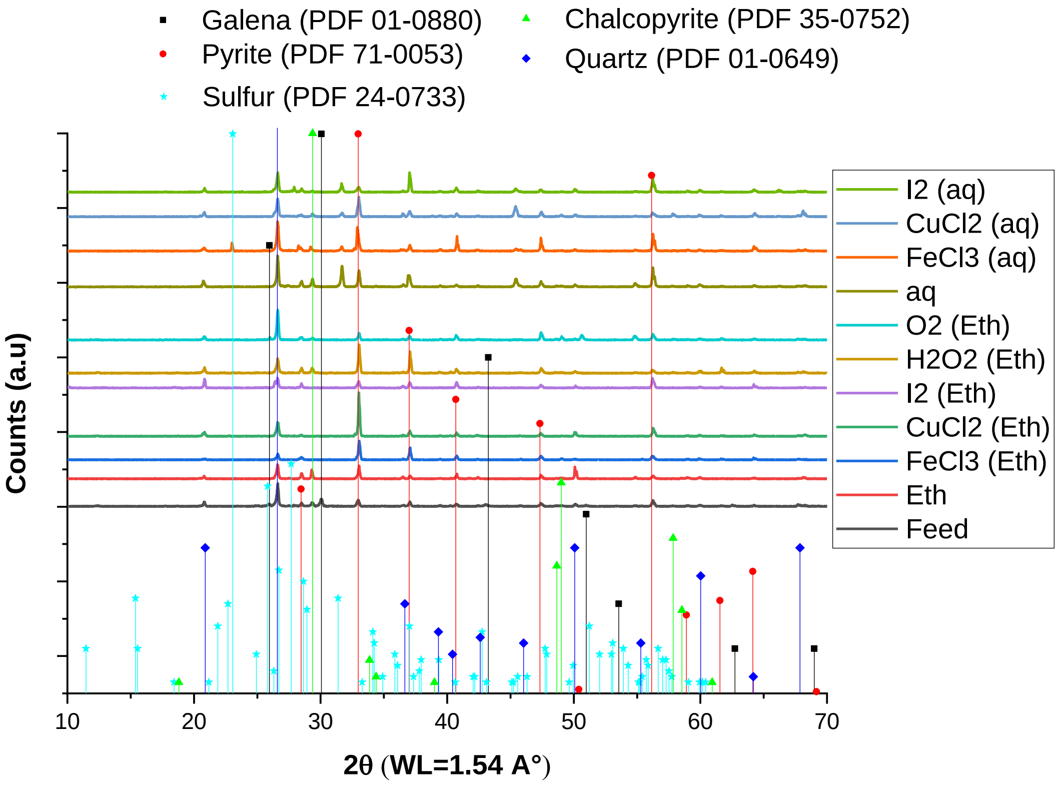

Fig. S1- XRD pattern of feed sample and residues after 24hr leaching in Ethaline at 80°C.

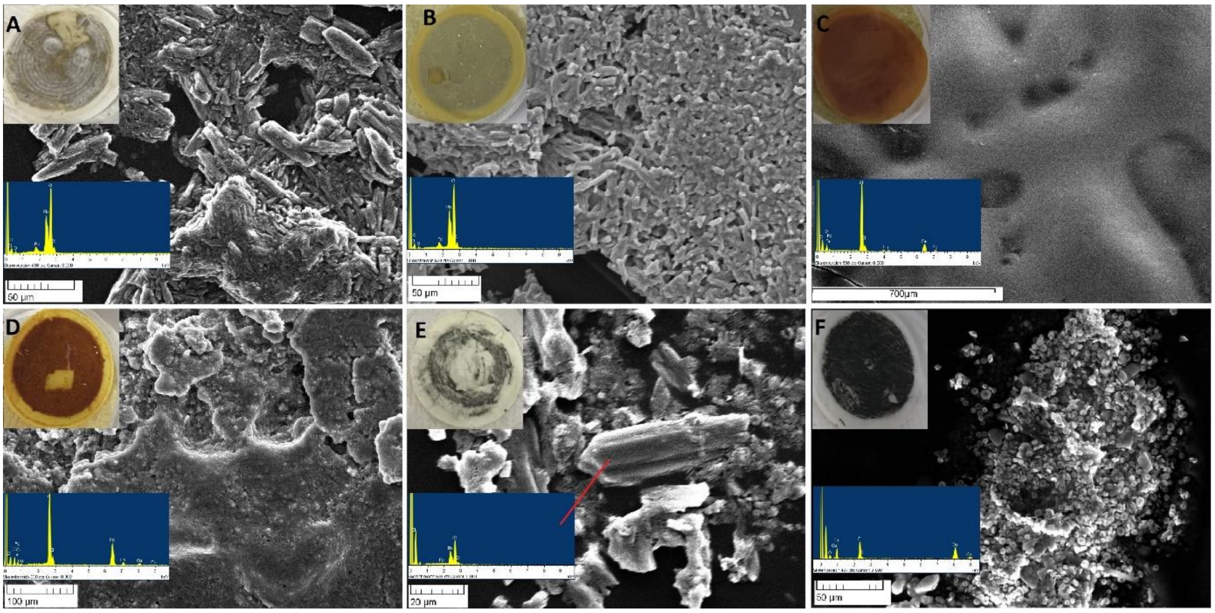

Fig. S2- SEM/EDX analysis of solids precipitated from leaching solution after a week. A: Ethaline (No additive), B: Ethaline+0.2M-$FeCl_3$, C: Ethaline+0.1M-I_2, D: Ethaline+0.2M-$CuCl_2$, E: Ethaline+0.2M-H_2O_2, F: Ethaline+O_2.

Fig S2 shows the SEM images and EDX analyses of the precipitates. Long rods of $PbCl_2$ were identified as the main precipitate in Ethaline, Ethaline+0.2M-$FeCl_3$ and Ethaline+0.2M-H_2O_2 systems that can be distinguished by their white colors. Brown Fe oxide was detected in Ethaline+0.1M-I_2 and Ethaline+0.2M-$CuCl_2$ systems. Minor amounts of Cu was detected in the latter case that can be attributed to solution absorption on solid particles. Black copper oxide precipitates were identified in the presence of H_2O_2 and O_2.
